# Grit, Resilience, Mindset, and Academic Success in Physical Therapist Students: A Cross-Sectional, Multicenter Study

**DOI:** 10.1093/ptj/pzac038

**Published:** 2022-04-11

**Authors:** Marlena Calo, Belinda Judd, Lucy Chipchase, Felicity Blackstock, Casey L Peiris

**Affiliations:** Department of Physiotherapy, School of Allied Health, Human Services and Sport, La Trobe University, Melbourne, Australia; Department of Physiotherapy, Faculty of Medicine and Health, University of Sydney, Sydney, Australia; Department of Physiotherapy, College of Nursing and Health Sciences, Flinders University, Adelaide, Australia; Department of Physiotherapy, School of Health Sciences, Western Sydney University, Sydney, Australia; Department of Physiotherapy, School of Allied Health, Human Services and Sport, La Trobe University, Melbourne, Australia

**Keywords:** Resilience, Mindset, Health Professional Students, Grit, Clinical Education, Academic Success

## Abstract

**Objective:**

The purpose of this study was to determine the relationships between noncognitive traits (grit, resilience, and mindset-type), academic success, and clinical performance in physical therapist students.

**Methods:**

This cross-sectional study using self-administered surveys was undertaken with final-year physical therapist students enrolled in 4 Australian universities. Participants completed validated questionnaires measuring grit, resilience, and mindset type. Academic transcripts were obtained to quantify academic success and clinical performance. A multiple regression analysis explored predictors of academic success and clinical performance in relation to sociodemographic factors, grit, resilience, and mindset type.

**Results:**

A total of 266 students participated in the study (80% recruitment rate). Overall, 25% of students had low resilience, 20% had low grit, and 14% had a fixed mindset type. Grittiness was positively associated with academic success (*r* = 0.24) and clinical performance (*r* = 0.22) and negatively associated with failing a clinical placement (*r* = ‐0.20). Grit was an independent predictor of overall academic success (β = 0.24, *P* ≤ .01) and clinical performance (β = 0.15). Students with low grit were twice as likely to fail a clinical placement compared with students with moderate or high grit (risk ratio = 2.03, 95% CI = 1.06 to 3.89).

**Conclusion:**

Grit was an independent predictor for overall academic success and clinical performance in final-year physical therapist students. Low grit may impact negatively on learning and students’ ability to cope with challenges associated with university studies and clinical education. Further studies should investigate interventions that best develop grit in health professional students and the overlapping nature of grit, resilience, and a growth mindset.

**Impact:**

This study helps universities and educators understand noncognitive factors predicting academic success and clinical performance in physical therapist students. Universities and clinical educators may consider screening and providing proactive strategies for students with low grit to improve success and general wellbeing.

## Introduction

Grit, resilience, and mindset type are increasingly recognized as noncognitive traits underpinning academic success and well-being.^1–3^ Resilience is commonly cited as a critical 21st-century graduate capability.^4^ Physical therapy is a demanding, hands-on university training program where students face physical, emotional, and cognitive challenges as they prepare for, transition into, and navigate the complex and often unpredictable clinical learning environment.^5–7^ Some students thrive on the challenges whereas others barely survive. Understanding factors predicting this variance may help universities, academics, and clinical educators to identify and proactively support students who may struggle.

Noncognitive traits are factors related to personality, temperament, and attitude, broadly defined as differences in individual tendencies to show consistent patterns of thought, feelings, and behavior.^8^ The increasing focus on traits of grit, resilience, and mindset type in educational research developed from pioneering work in school-age children demonstrating grit^9^ and mindset,^10^^,^^11^ rather than intelligence alone, played an important role in predicting academic success and well-being. Grit is defined as perseverance and passion towards longer-term goals and sustained commitment to completing an endeavor despite episodes of failure, setbacks, and adversity.^9^ Resilience, although having multiple definitions, is defined for health professionals as “the dynamic capacity to overcome adversity, drawing on personal, social and organizational resources, to achieve personal growth and transformation.”^4^ Mindset type is defined as the degree to which an individual believes that one’s intelligence or ability is changeable, with one end of the spectrum referred to as a fixed mindset and the opposing end referred to as a growth mindset.^12^^,^^13^ For example, an individual who believes one’s intelligence cannot be altered in a meaningful way has a fixed mindset in relation to intelligence. Conversely, a growth-mindset individual believes intelligence can be improved over time with effort and dedication.^14^

Clinical training is an essential component in physical therapy programs, with the average Australian student undertaking anywhere between 800 and 1200 hours of clinical education during their training program.^15^ The transition from classroom-based learning to the clinical learning environment is a time of increased stress as students translate theoretical knowledge into real-world practice.^7^^,^^16^ Health professional students with low levels of grit, resilience, and/or a fixed-mindset are at risk of increased perceived stress because they may lack the ability to positively adapt to feedback, bounce back, and persevere with challenges and setbacks.^17^^,^^18^ Preliminary research in physical therapist students at 1 university found 25% had low resilience and 13% had low grit, suggesting that 1 in 4 students may be at higher risk of stress and poorer coping ability when faced with challenges.^19^ This is a concern for universities, academics, and clinical educators, as stress and poor coping may interfere with effective learning, clinical performance, and the capacity to care for patients.^16^ Understanding the relationship between grit, resilience, and mindset type and academic success, particularly clinical performance, in physical therapist students may assist with early identification of students at risk of poor performance.

Research exploring the importance of grit, resilience, and mindset type in health professional students has increased substantially over the past decade, and multiple recent scoping reviews exist.^2–4^ The majority of studies have been undertaken in medical, nursing, pharmacy, and dentistry disciplines; a recent systematic review explored the correlation of grit and resilience with academic success, yielding mixed results with a general trend towards a positive association.^1^ To date, only 1 larger study has investigated the association between grit and academic success in physical therapist students, finding a positive association in 168 entry-level doctor of physical therapy students in America.^20^ No studies have explored the relationship between resilience and academic success in physical therapist students. To the authors’ knowledge, no literature has investigated the relationship between mindset type and academic success in health professional students; however, there were no relationships between the 2 constructs in veterinary students.^21^ Because scant studies have explored the relationship between grit and academic success in physical therapist students and no studies have explored clinical placement performance or the relationship between resilience and/or mindset type and academic success in physical therapist students, the primary aims of this research were to investigate if grit, resilience, and/or mindset type were related to overall academic success, clinical performance, or failing a clinical education course/unit of study. The secondary aims were to (1) determine relationships between sociodemographic factors and grit, resilience, mindset type, and/or academic success in physical therapist students; and (2) determine relationships between grit, resilience, and mindset-type.

## Methods

This cross-sectional study was conducted at 4 universities in Australia between December 2018 and December 2019. The study was approved by the human research and ethics committees at each site (H13024, HEC18431, Project ID 1649). The Strengthening Reporting of Observational Studies in Epidemiology statement was used to ensure the rigor of reporting.^22^

### Recruitment

All final-year pre-registration physical therapist students at the 4 universities were invited to participate via email. In Australia, eligibility to practice as a physical therapist can be obtained via a number of different pathways, which all meet the same accreditation standards (undergraduate degrees: bachelor, combined bachelor/masters, or doctor of physical therapy; or postgraduate master’s degree). Of the 4 universities in this study, all offered undergraduate degrees (bachelor or combined bachelor/masters) and 2 also offered postgraduate master’s degrees. Students were recruited within the final 3 months of completing their physical therapy training program. Final academic results were obtained once all courses/units of study in the entire curriculum were completed. All participants provided written informed consent prior to completing the 8-minute survey.

### Data

The following data were collected through a paper-based survey:

1. Sociodemographic details, including age, gender, diagnosed mental health condition, disability, living arrangements, course type (undergraduate or postgraduate) and hours per week spent studying, working in paid employment, undertaking caring duties, and playing sports (Suppl. Data 1). Sociodemographic questions were chosen based on factors associated with psychological distress in university students.^23^
2. Grit was measured via the Grit-S scale, a validated 8-item scale.^24^ Predictive validity, consensual validity, and test–retest reliability of the Grit-S questionnaire was found to be good when measured against other commonly utilized tools such as the Grit-O and the Big 5 Personality across the lifespan in United States military academy cadets (n = 2526), Ivy League college students (n = 134), and national spelling bee finalists (n = 175), with the average Grit-S scores in these populations ranging from 3.46 to 3.78.^24^
3. Resilience was measured via the Brief Resilience Scale (BRS), a validated 3-item scale,^25^ and the Academic Resilience Scale (ARS), a validated 30-item scale.^26^ Good internal consistency, convergent validity, and discriminant predictive validity of the BRS were found when measured against several other resilience scales in a sample of 192 undergraduate students, 112 cardiac rehabilitation clients, and 50 women with fibromyalgia.^25^ Normative data showed an average BRS score of 3.53 out of 5 (SD 0.68).^25^ The ARS demonstrated good internal reliability and construct validity in a sample of 532 British undergraduate students compared with the General-Academic Self-Efficacy Scale, with the average ARS value reported as 115.61 (SD 14.78).^27^
4. Mindset type was measured by the Dweck Mindset Instrument (DMI), a 16-item scale (Dweck 1999) adapted from the reliable 8-item Implicit Theory of Intelligence Questionnaire.^28^ The DMI has 2 subsections, which score mindset in relation to intelligence (DMI-I) and talent (DMI-T) respectively. Internal reliability and confirmatory factor analysis were acceptable (Cronbach α >70) in 232 secondary year students; however, despite the frequent use of this tool, normative data are not available.^27^

The following data were exported electronically from individual academic transcripts downloaded from the 4-university student academic management systems:

1. Overall academic success was measured by the weighted average mark (WAM) out of 100 for the entire curriculum undertaken in the program. These included all classroom-based and clinical environment-based courses/units of study. The WAM is calculated by considering each course/unit of study, which is weighted according to the credit points associated with that course/unit of study.
2. Clinical performance was measured by calculating the average mark out of 100 of all courses/units of study completed in the curriculum undertaken and assessed in the clinical environment by a clinical educator, referred to as a “clinical placement.” According to credit points, all clinical placements were weighted equally at each university. A failed clinical placement was classified as score <50%.

### Data Collection

Grit, resilience, mindset type, and sociodemographic measures were collected from students during class through a paper-based survey in the final 3 months of completing their physical therapy training program in an attempt to standardize assessment time.

Academic transcripts were accessed retrospectively 3 months post survey by the research team to calculate overall academic success and clinical performance.

### Statistical Analysis

Means, SDs, and frequencies were calculated using IBM Statistical Product and Service Solutions (SPSS, Armonk, NY, USA) version 27^29^ to describe the sample. Missing data were excluded from analysis. Relationships between grit, resilience, mindset type, sociodemographic factors, overall academic success, clinical performance, and failing a clinical placement were first analyzed using bivariate correlations for continuous variables and point biserial correlations for a categorical variable (eg, gender) and a continuous variable. Continuous scores for Grit-S, ARS, BRS, DMI-I, and DMI-T were used to determine correlations. The strength of the correlation was considered very strong if r ≥ ±0.9; strong if r = 0.7 to 0.98; moderate if r = 0.5 to 0.69; weak if r = 0.3 to 0.49; and little or no correlation if r < ±0.3.^30^

Relative risk ratios (RR) and 95% CIs were calculated using categorical data for Grit-S, ARS, BRS, DMI-I, and DMI-T to determine relationships between 2 categorical variables (eg, female and low grit or failed and low resilience). Data were dichotomized for RR calculations to compare one “category” of students (eg, low grit) with all other students (ie, those with moderate and high grit). The BRS and DMI have predefined categorical ratings (low, moderate, and high) based on scores (Tab. 1).^14^^,^^25^ The ARS and Grit-S do not have predefined categorical ratings based on scores. For interpretability, we categorized the Grit-S as per the BRS because both are 5-point Likert scales with average scores calculated to obtain a score between 1 to 5 (Tab. 1). We classified “normal” academic resilience as the mean normative value ±SD. Normative data of university students show the average ARS value was 115.61 (SD 14.78).^28^ Therefore, students were categorized as having moderate academic resilience when their ARS score was between 100.83 and 130.39 (ie, 115.61 ± 14.78), as having low academic resilience if ARS < 100.83, and high academic resilience if ARS > 130.39 (Tab. 1).

**Table 1:** Validated Surveys of Grit, Resilience, and Mindset Type*^a^*

**Survey**	**Items**	**Scoring**	**Categories**
Grit-S	8 Items on a 5-point Likert scale	Scores are averaged to obtain a maximum score between 1 (not at all gritty) and 5 (extremely gritty)	1–2.99: low grit 3–4.3: normal grit 4.31–5: high grit
BRS	6 Items on a 5-point Likert scale	Scores are averaged to obtain a score between 1 (lowest resilience) and 5 (highest resilience)	1–2.99: low resilience 3–4.3: normal resilience
			4.31–5: high resilience
ARS-30	30 Items on a 5-point Likert scale	Scores are summed (theoretical range 30–150), with a higher global score reflecting greater academic resilience	<100.83: low academic resilience 100.83–130.39: moderate academic resilience
			>130.39: high academic resilience
DMI	16 Items on a 6-point Likert scale	Two subsets are measured: intelligence (DMI-I items 1–8) and talent (DMI-T items 9–16). For each subset, scores are averaged to give a final score between 1 (fixed mindset) and 6 (growth mindset) for each	1–3: fixed trait 3.1–3.9: undecided 4–6: malleable (growth) trait

^a^ ARS = Academic Resilience Scale; BRS = Brief Resilience Scale; DMI = Dweck Mindset Instrument; GRIT-S = Short Grit Scale.

A standard multiple regression analysis was used to determine predictors of overall academic success and clinical performance (dependent variables) using a backwards elimination process starting with all predictor variables (grit, resilience, mindset type, and sociodemographic factors) expected to contribute. Continuous scores were used for Grit-S, ARS, BRS, DMI-I, and DMI-T. Categorical variables (eg, age, gender, living arrangements) were recoded to be dichotomous (eg, female or not female, >24 years old or not >24 years old, lives with family or does not live with family). Theory marks were determined as the weighted average mark of all classroom-based courses/units of study (eg, overall academic performance excluding all clinical environment-based courses/units of study) and were used as a predictor variable for clinical performance. Pearson product correlation was first used to assess any association between predictor variables (collinearity). Variables were removed from the model if there was more than a weak correlation present (r > 0.49).^30^ Final prediction models were reached when all predictor factors were statistically significant (*P* < .05).

To determine the sample required for the multiple regression analysis, the following formula was used: n > 50 + 8 m, where m is the number of independent contributing factors and n is the number of participants needed.^30^ Based on this formula, the estimated sample size required was 170 (with 15 independent factors per analysis).

## Results

**Figure 1:**
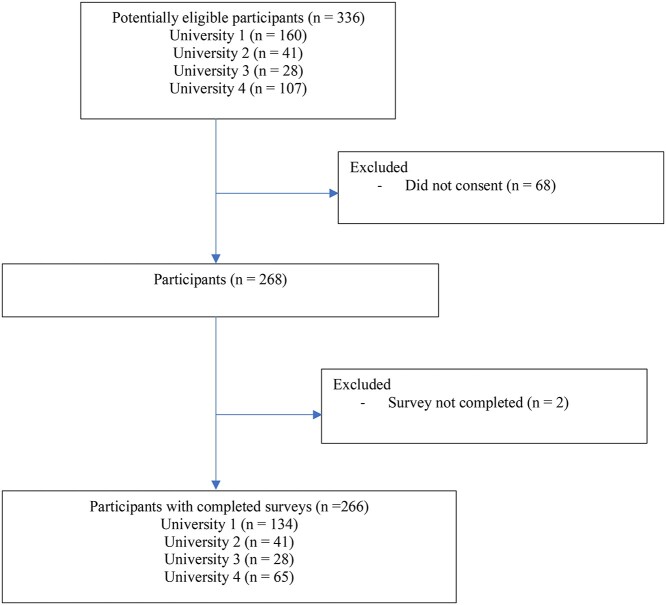
Flow of participants through the study.

A total of 336 final-year physical therapist students were invited to participate, and 268 students volunteered (recruitment rate 80%) (Fig. 1).

### Descriptive Data

Of students completing the survey, 55% were female and aged between 22 and 24 years, and 51% were living at home with parents/family. One in 5 were international students, and 1 in 5 reported a diagnosed mental health disorder (Tab. 2). The mean WAM was 76.5 (SD 5.6) and the mean clinical mark was 76.7 (SD 7.7). Twelve percent of students (n = 32) failed a clinical placement. There were no statistically significant differences between universities in terms of student academic or clinical performance; hence, data are reported as a whole data set.

**Table 2:** Participant Sociodemographics

	**Total (n = 266)**	
**Characteristic**	**No.**	**%**
Gender		
Male	111	41.7
Female	146	54.9
Nonbinary, fluid, queer	9	3.4
Age, y*^a^*		
19–21	39	14.7
22–24	145	54.5
25–27	53	19.9
28–30	14	5.3
31+	11	4.1
Course*^a^*		
Postgraduate (master’s)	65	24.4
Undergraduate	197	74.1
Disability	21	7.9
Diagnosed mental health condition	54	20.3
International student	48	18
Carer for family member >5 h/wk	72	27
Living arrangements*^a^*		
On campus	3	1.1
With domestic partner	32	12
With parents/family	136	51.1
Sharing with friends/flatmates	75	28.2
Living alone	10	3.8
Other	2	.8
Playing sport, h/wk*^a^*		
<5	116	43.6
5–9	88	33.1
10–14	37	13.9
15–19	13	4.9
20+	7	2.6

^a^ Data are missing; do not add up to 100%.

Mean scores and categorical frequencies of grit, resilience, and mindset type are reported in Table 3. Overall, 25% of students had low academic resilience, 20% of students had low grit, 14% had a fixed mindset in relation to talent and 10% in relation to intelligence.

**Table 3:** Grit, Resilience, and Mindset-Type Scores and Categories*^a^*

				**Categories, no. (%)**		
**N = 266**	**Min**	**Max**	**Mean (SD)**	**Low**	**Moderate/normal**	**High**
Grit-S (n = 264)	1.75	4.88	3.49 (.64)	51 (19.2%)	184 (69.2%)	29 (10.9%)
BRS (n = 260)	1	5	3.40 (.75)	61 (22.9%)	170 (63.9%)	29 (10.9%)
ARS (n = 265)	51	147	110.34 (15.47)	66 (24.8%)	175 (65.8%)	24 (9.0%)
				**Fixed**	**Undecided**	**Growth**
DMI-I (n = 264)	1	6	4.39 (0.95)	26 (9.8%)	54 (20.3%)	184 (69.2%)
DMI-T (n = 264)	1	6	4.24 (1.01)	38 (14.3%)	59 (22.2%)	167 (62.8%)

^a^ ARS = Academic Resilience Scale; BRS = Brief Resilience Scale; DMI-I = Dweck Mindset Instrument (Intelligence); DMI-T = Dweck Mindset Instrument (Talent); Grit-S = Short Grit Scale.

### Relationships Between Noncognitive Traits and Academic Success, Clinical Performance, Failing a Clinical Placement, and Sociodemographics

Relationships between grit, resilience, and mindset type and overall academic success, clinical performance, and sociodemographic factors are reported in Table 4 and Supplementary Data 2.

**Table 4:** Correlations Between Sociodemographic Factors and Grit, Resilience, and Mindset*^a^*

	**International**	**Postgraduate**	**Sport <5 h/wk)**	**Overall Academic Success**	**Clinical Performance**	**Failing a Clinical Placement**	**Gender (LGBT IQ+ vs Male/Female)**	**Gender (Male vs Female)**
Grit-S	−0.165*^b^* (*P* = .008)	0.104 (*P* = .090)	−0.150*^c^* (*P* = .015)	0.239*^b^* (*P* < .001)	0.220*^b^* (*P* < .001)	−0.198*^b^* (*P* = .001)	−0.032 (*P* = .601)	−0.024 (*P* = .705)
BRS	−0.043 (*P* = .490)	0.076 (*P* = .220)	−0.167*^c^* (*P* = .007)	0.011 (*P* = .867)	−0.015 (*P* = .811)	−0.114 (*P* = .065)	−0.017 (*P* = .780)	0.087 (*P* = .164)
ARS	−0.001 (*P* = .993)	0.067 (*P* = .067)	−0.187*^b^* (*P* = .002)	0.058 (*P* = .351)	0.033 (*P* = .593)	−0.099 (*P* = .107)	−0.051 (*P* = .410)	−0.065 (*P* = .297)
DMI-I	−0.209*^b^* (*P* = .001)	0.127*^c^* (*P* = .039)	−0.210*^b^* (*P* = .001)	0.026 (*P* = .671)	0.083 (*P* = .184)	−0.043 (*P* = .485)	−0.002 (*P* = .972)	−0.082 (*P* = .189)
DMI-T	−0.161*^b^* (*P* = 009)	0.175*^b^* (*P* = .004)	−0.148*^c^* (*P* = .016)	−0.071 (*P* = .251)	0.074 (*P* = .233)	−0.061 (*P* = .322)	−0.105 (*P* = .089)	−0.087 (*P* = .161)

^a^ Raw scores. See Supplementary Data 2 for complete table. ARS = Academic Resilience Scale; BRS = Brief Resilience Scale; DMI-I = Dweck Mindset Instrument (Intelligence); DMI-T = Dweck Mindset Instrument (Talent); Grit-S = Short Grit Scale.

^b^ Correlation is significant at the 0.01 level (2-tailed).

^c^ Correlation is significant at the 0.05 level (2-tailed).

### Academic Success and Clinical Performance

Grit was positively correlated with academic success (r = 0.24, *P* ≤ .001) and clinical performance (r = 0.22, *P* = .001) (Tab. 4). Resilience and mindset type were not correlated with overall academic success or clinical performance.

### Failing a Clinical Placement

Traits that significantly increased the risk of failing a clinical placement are presented in Table 5. Compared with students with moderate or high grit, students with low grit were twice as likely to fail a clinical placement. Students with a disability, a diagnosed mental health condition, international students, males, and LGBTIQ+ students were all significantly more likely to fail a clinical placement.

**Table 5:** Risk Ratio (95% CI) for Risk of Failing a Clinical Placement*^a^*

**Trait**	**Risk Ratio**	**95% CI**	***P***
Low grit	2.03	1.06 to 3.89	.032
Disability	2.51	1.27 to 4.92	.018
Mental health condition	2.05	1.05 to 4.00	.034
International	2.58	1.17 to 5.65	.008
Male	3.12	1.42 to 6.87	.005
LGBTIQ+	5.29	2.67 to 10.49	<.001

^a^ LGBTIQ+ = lesbian, gay, bisexual, trans, intersex, queer/questioning.

There was a weak negative correlation between grit and failing a clinical placement (r = ‐0.20, *P* = .001). Resilience and mindset type were not correlated with failing a clinical placement (Tab. 4).

### Sociodemographic Factors

There was a weak negative correlation between playing sport for <5 h/wk and grit (r = −0.15, *P* = .015), resilience (r = −0.19, *P* = .002), and a growth mindset for intelligence (−0.21, *P* = .001) and talent (r = −0.15, *P* = .016).

Being a postgraduate student was weakly positively correlated with a growth mindset for intelligence (r = 0.13, *P* = .039) and talent (r = 0.175, *P* = .004).

Being an international student was weakly negatively correlated with both grit (r = −0.17, *P* = .008) and a growth mindset for intelligence (−0.21, *P* = .001) and talent (−0.16, *P* = .009). International students were twice as likely to be categorized as having low grit (RR 2.08, 95% CI = 1.27 to 3.41, *P* = .004).

### Grit, Resilience, and Mindset Relationship to Each Other

Compared with students with moderate or low grit, students with high grit were 4 times more likely to have high resilience as measured by the ARS (RR = 4.10, 95% CI = 1.09 to 15.86, *P* = .038) and 38% more likely to have a growth mindset in relation to intelligence (RR = 1.38, 95% CI = 1.04 to 1.80, *P* = .022).

### Predictors of Overall Academic Success and Clinical Performance

When checking for violations of assumptions, significant collinearity was detected between ARS and BRS and between DMI-I and DMI-T talent scores. The measures of ARS and DMI-I were chosen to be retained in the analysis because they were stronger predictors of the dependent variables. Average scores of grit, resilience, and mindset type did not statistically differ between universities; hence, the data were investigated as a whole.

#### Overall Academic Success

The final standard linear regression model found sociodemographic and grit scores predicted 43% of the variance in overall course WAM (R^2^ = 0.43, F = 14.21, *P* < 0.001). Having a higher grit score (β = .24) and being female (β = 0.23) contributed positively to the model, whereas being a postgraduate student (β = −.18) and having a diagnosed mental health condition (β = −.18) contributed negatively (Tab. 6).

**Table 6:** Multiple Regression Analysis for Predictors of Overall Academic Success and Clinical Performance*^a^*

**Variables**	**B**	**95% CI for B**	**β**	***t***	***P***
Overall academic success^***b***^					
Independent variables included in final model					
Grit (Grit-S)	2.03	1.05 to 3.01	.24	4.11	.000
Female	2.52	1.29 to 3.77	.23	3.98	.000
Mental health	−2.29	−4.01 to −0.92	−.18	−3.15	.002
Postgraduate	−2.47	−3.74 to −0.84	−.18	−3.10	.002
Independent variables not included in final model					
Lives with parents	−1.43	−2.92 to 0.05	−.13	−1.90	.058
Resilience (ARS)	−0.02	−0.06 to 0.03	−.05	−0.69	.488
Mindset (DMI-I)	0.02	−0.71 to 0.75	.00	0.04	.965
Age	0.36	−1.22 to 1.94	.03	0.45	.654
Disability	0.23	−2.30 to 2.75	.01	0.18	.858
Study <5 h/wk	−0.23	−2.92 to 2.36	−.01	−0.21	.836
Paid employment	−1.51	−3.58 to 0.56	−.09	−1.45	.152
Sport <5 h/wk	0.27	−1.13 to 1.67	.02	0.38	.703
Carer	−0.07	−1.57 to 1.42	.00	−0.10	.923
International	−0.98	−2.94 to 0.98	−.07	−0.99	.326
Clinical performance*^c^*					
Independent variables included in final model					
Female	2.13	0.41 to 3.85	.14	2.43	.016
Grit (Grit-S)	1.81	0.48 to 3.13	.15	2.68	.008
Theory marks	0.50	0.34 to 0.67	.34	5.91	.000
Disability	−5.59	−8.53 to −2.63	−.21	−3.73	.000
Lives with parents	−2.04	−3.71 to −0.36	−.13	−2.40	.017
Independent variables not included in final model					
Postgraduate	1.96	−0.26 to 4.18	.11	1.74	.083
Mental health	−0.33	−2.61 to 1.96	−.17	−0.28	.782
Resilience (ARS)	−0.04	−0.10 to 0.03	−.07	−1.11	.269
Mindset (DMI-I)	0.24	−0.73 to 1.22	.03	0.50	.621
International	−0.88	−3.48 to 1.73	−.04	−0.66	.508
Age	0.62	−1.47 to 2.72	.04	0.59	.558
Study <5 h/wk	1.50	−2.00 to 5.00	.05	0.85	.399
aid employment	1.87	−0.88 to 4.64	.08	1.34	.182
Sport <5 h/wk	−1.07	−2.93 to 0.79	−.07	−1.14	.257
Carer	−0.06	−2.04 to 1.92	−.03	−0.06	.954

^a^ ARS = Academic Resilience Scale; B = unstandardized coefficient; Beta = standardized coefficient; DMI-I = Dweck Mindset Instrument (Intelligence); Grit-S = Short Grit Scale.

^b^ R^2^ = 0.43; F = 14.21; *P* < .001.

^c^ R^2^ = 0.50; F = 16.83; *P* < .001.

#### Clinical Performance

The final standard linear multiple regression model found sociodemographic and grit scores predicted 50% of the variance in clinical performance (R^2^ = 0.50, F = 16.83, *P* < .001). Average theory mark (β = .34), having a higher grit score (β = .15), and being female (β = .14) contributed positively to clinical performance. Having a disability (β = −.21) and living at home with family/parents (β = −.13) contributed negatively to clinical performance (Tab. 6).

## Discussion

In 266 physical therapist students across 4 universities, grit was an independent predictor of overall academic success and clinical performance, accounting for 24% and 15% of the variance, respectively. Students categorized with low grit were 2 times more likely to fail a clinical placement compared with students with moderate to high grittiness. Our study of physical therapist students from undergraduate and entry-level postgraduate (masters) programs in Australia supports the findings of a positive linear relationship between grit and academic success also found in 168 physical therapist students from 1 entry-level postgraduate (doctor of physical therapy) program in America.^20^ A positive correlation between grit and academic success has also been reported in medical, pharmacy, and dentistry students.^31–33^ In addition to overall academic success, this study is the first, to our knowledge, to show a direct relationship between grit, clinical performance, and risk of failing a clinical placement in physical therapist students.

Grit is a measure of persistence to completing endeavors despite episodes of failure and setbacks.^9^ Physical therapist students face many common challenges and setbacks throughout their studies that require persistence. The dynamic clinical learning environment in particular presents many obstacles as students are challenged physically, emotionally, and cognitively in an environment with exposure to patient suffering and death, and students commonly experience compassion fatigue.^16^^,^^34^ In conjunction with clinical stressors, students balance the everyday personal, relationship, and financial stressors of transitioning into adulthood.^35^ Despite common challenges and setbacks, how an individual responds to challenges will affect their ability to thrive or simply survive. The variance in grit levels found in this study helps to understand how different students may respond to challenges and the impact on performance. For example, a student with low grit may struggle to positively adapt to setbacks and may lack the persistence needed to sustain the effort required to overcome challenges in the learning environment, resulting in lowered levels of academic success and clinical performance.^18^

Pre-admission testing consisting of written tests, surveys, and/or interviews to test cognitive and noncognitive traits for entry into health professional programs is common practice in many countries.^36^^,^^37^ Adopting grit measures into pre-admission testing in combination with other noncognitive traits such as emotional intelligence, resilience, and psychological flexibility has been suggested to promote selection of holistic applicants into physical therapy programs.^38^ Pre-admission testing is not common practice in Australian physical therapy programs and was not utilized by universities in this study. The use of grit measures in pre-admission selection may screen out potentially good candidates who may not have been exposed to sufficient life experiences to face challenges required to develop grit. Grit generally develops with age,^18^ and, interestingly, living at home with parents was an independent predictor for lowered clinical performance in our study. This may be because students living at home may not have faced the challenges of living in the real world and are likely to be younger (which many students would be at pre-admission screening). The average level of grit and resilience of final-year physical therapist students in this study was similar to normative data for high-achieving secondary students for the Grit-S^24^ and undergraduate students for the BRS^25^ and ARS^28^; however, how students’ grit and resilience levels changed from the commencement of study is unknown. Therefore, instead of pre-admission screening of grit being used to exclude or select students for admission, it could be utilized to identify and support students at higher risk of poorer academic success and/or clinical performance.

One in 5 physical therapist students had low levels of grit and 1 in 4 had low levels of resilience in our study, leaving them exposed to increased risk of poor performance due to decreased ability to cope with challenging situations. The Grit-S and BRS tools are quick and easy to administer with 8 and 6 survey items, respectively. Screening may be implemented at the commencement of study; however, as previously described, many students at this time point may not yet have experienced sufficient challenges to develop grit and/or resilience. Screening at a time point further into the program may allow for exposure to some challenges and more accurately identify students at risk. A potentially critical time point where screening may ideally occur is prior to commencing clinical placements. The transition from classroom-based learning to the clinical environment is a time of increased stress as students face multiple new challenges in the dynamic, real-world environment.^16^^,^^39^ Because students with low grit were twice as likely to fail a clinical placement in this study, it is conceivable that students with low grit may cope poorly with additional challenges in the clinical environment, which could impact both student and patient safety. Hence, screening of grit within physical therapy programs may be most useful prior to commencement of clinical placements.

In this study, grit was a significant independent predictor of academic success and clinical performance. Although resilience and a mindset type were not found to be significant independent predictors of student performance, we cannot disregard the importance of these 2 traits because they are inter-related with grit^18^ and a range of other important noncognitive traits such as locus of control, emotional intelligence, the big 5 personality traits (openness, conscientiousness, extraversion, agreeableness, and neuroticism) and perceived stress.^1^^,^^36^ The interplay between grit, resilience, a growth mindset, and other noncognitive traits is poorly understood; for example, resilience is thought to be an attribute of grit and is intimately related to a growth mindset.^26^^,^^40^ Results of this study support this concept of interrelatedness as students with high levels of grit were 4 times more likely to have high resilience and 38% more likely to have a growth mindset compared with those with moderate or low levels of grit. Because of this overlap, we should not solely focus on 1 trait in isolation because we risk losing a deeper understanding of the complexities at play. Further understanding of how grit, resilience, a growth mindset, and other noncognitive traits interrelate may help guide theoretical models and interventions to support academic success and well-being of health professional students.

Potential interventions to increase grit, resilience, and/or a growth mindset in health professional students are not well established and require further investigation.^2^^,^^40^ In the absence of clear evidence-based strategies, the current practical focus for academics and clinical educators should be on promoting supportive learning environments for all students and addressing any modifiable individual factors for students at high risk of poorer academic success and/or clinical performance. For students at high risk, individual learning plans can be developed and referrals made to relevant university support services such as counseling, financial, international, disability, and study skills services. A degree of stress and challenge is important in learning, and grit and resilience develop through experiencing and overcoming obstacles.^41^ Hence, the aim of implementing supports for high-risk students and creating supportive learning environments for all students is not to diminish challenges, but to assist students to positively adapt to common day-to-day stressors faced by all health professional students throughout their studies. Creating supportive learning environments involves considering the design and scaffolding of curriculum and design of assessments to include student autonomy and embedding a sense of belonging.^42^ These organizational approaches are in line with key factors reported in an Australian study of 4000 university students, whereby students cited these factors as key to improving their well-being and resilience.^43^

The primary aim of this paper was to investigate the association between grit, resilience, and mindset type on academic success and clinical performance. It is also important to consider other factors impacting on academic success such as socio-demographics. This study found having a diagnosed mental health condition and/or being an entry-level postgraduate (masters) student were predictive of lowered overall academic success, and having a disability was a predictor of lowered clinical performance. In addition, students with a diagnosed mental health condition, disability, male students and international students were all more than twice as likely to fail a clinical placement. This study found LGBTIQ+ students were more than 5 times more likely to fail a clinical placement. Although low numbers (n = 9) preclude strong conclusions, this finding is concerning because we also know a disproportionate number of LGBTIQ+ people have poorer mental health outcomes, experience higher risk of suicidal behaviors, and experience high levels of stigma and discrimination compared with the general population.^44^ Universities should ensure programs are available to enhance inclusion of LGBTIQ+ students, including peer-led programs, provision of gender-neutral toilets, increasing queer visibility, and ensuring students have access to appropriate mental health services and sexual health information.^45^ Further research is warranted to understand how to best support LGBTIQ+ students in physical therapy. Diversity within the physical therapy profession must be supported by ensuring all high-risk students are considered and offered proactive supports to maximize the likelihood of academic success and well-being.

### Limitations

Methodological strengths of this multi-center research study include a high response rate and standardization of the time point participants undertook the surveys. Limitations include the use of self-reported measures for grit, resilience, and mindset type, which may be subject to the effects of social desirability; however, no objective measures currently exist. The correlations found in this study were weak; however, conclusions were drawn from all results, with a particular focus on regression analysis findings and RRs. There are limitations to cross-sectional studies, and future longitudinal studies across year levels would be beneficial. Although anxiety and depression were reported on, this work could be expanded to investigate other variables such as perceived stress levels. The transition from student to new graduate physical therapist is another steep learning curve, where new graduates navigate the work environment, increased complexity of patients, higher caseloads, and understanding the healthcare system and experience confidence deficits surrounding clinical and professional skills.^46^ Further research investigating how grit, resilience, and mindset type impact on career and life success may help to develop holistic solutions to approaching the high attrition rate of early-career physical therapists.^15^

This study demonstrated grit was both an independent predictor of overall academic success and clinical performance in physical therapist students, and students with low grit were twice as likely to fail a clinical placement compared with those with moderate or high grit. One in 5 students had low levels of grit and 1 in 4 had low levels of resilience, which is of concern to both universities and clinical educators because these students are more likely to struggle with the stress and challenge of university studies, the clinical environment, and potentially the transition to entering the workforce. Further research is required to investigate the interplay between grit, resilience, and a growth mindset and best practice interventions at the individual and broader organizational levels to best develop these traits in health professional students and future new graduates.

## Supplementary Material

Supplementary_Appendix_1_pzac038

Supplementary_Appendix_2_pzac038
