# Supplementary material for: Grit, Resilience, Mindset, and Academic Success in Physical Therapist Students: A Cross-Sectional, Multicenter Study
Source: Phys Ther. 2022 Apr 11;102(6):pzac038. doi: 10.1093/ptj/pzac038 (PMC9350533; doi:10.1093/ptj/pzac038)
Supplement: Supplementary_Appendix_1_pzac038 [file supplementary_appendix_1_pzac038.pdf]

Supplementary Appendix 1: Demographic Survey

Please answer the following questions so we can establish trends in the data, keeping in mind that your answers will not identify you in any way.

What is your gender?: □ Female □ Male □ Non-binary; fluid; queer □ Prefer not to answer

What is your current age?: □ 19-21 □ 22-24 □ 25-27 □ 28-30 □ 31 +

Are you enrolled as an international student? □ Yes □ No

What physiotherapy course are you enrolled into?
□ Graduate Entry Masters Program
□ Bachelor of Applied Sciences and Master of Physiotherapy Program
□ Bachelor of Physiotherapy

Do you have disability that in some way affects your study? □ Yes □ No

Do you have a diagnosed mental health condition? □ Anxiety □ Depression □ Eating Disorder □ Other □ Not applicable

Where are you currently living? □ Uni residential college □ With domestic partner □ With parent/s or family members □ Sharing with friends/flatmates □ Living alone □ Other

Please answer the following questions by circling box that corresponds to your answer.

On average how, many hours per week do you spend studying outside class time?	Less than 5 hours	5-9 hours	10-14 hours	15-19 hours	20 hours or more
On average, how many hours per week do you work in paid employment during the semester?	Less than 5 hours	5-9 hours	10-14 hours	15-19 hours	20 hours or more
On average, how many hours per week do you participate in organised sport during the semester?	Less than 5 hours	5-9 hours	10-14 hours	15-19 hours	20 hours or more
On average, how many hours per week do you spend directly caring for family member/s?	Less than 5 hours	5-9 hours	10-14 hours	15-19 hours	20 hours or more

If you provide weekly care for family members, who are those family members (tick all that apply)? □ My own children □ Sibling children □ Adult family members □ Question Not applicable. Thank you for taking the time to complete this survey – it is very much appreciated!
