# Supplementary material for: Grit, Resilience, Mindset, and Academic Success in Physical Therapist Students: A Cross-Sectional, Multicenter Study
Source: Phys Ther. 2022 Apr 11;102(6):pzac038. doi: 10.1093/ptj/pzac038 (PMC9350533; doi:10.1093/ptj/pzac038)
Supplement: Supplementary_Appendix_2_pzac038 [file supplementary_appendix_2_pzac038.pdf]

Supplementary Appendix 2. Complete table of correlations between sociodemographic factors and grit, resilience and mindset (raw scores)

	International	Postgraduate	Sport <5hrs per week	Overall academic success	Clinical performance	Failing a clinical placement	Paid employment <5hrs per week	Age (<24yo vs >24yo)	Lives with parents/ family	Disability	Carer	Study time <5hrs per week	Gender (LGBTIQ+ vs male/female)	Gender (male vs female)
BRS	-0.043 (p=0.490)	0.076 (p=0.220)	-0.167* (p=0.007)	0.011 (p=0.867)	-0.015 (p=0.811)	-0.114 (p=0.065)	0.031 (p=0.616)	-0.072 (p=0.251)	-0.090 (p=0.147)	-0.029 (p=0.650)	-0.057 (p=0.356)	-0.083 (p=0.183)	-0.032 (p=0.601)	-0.024 (p=0.705)
Grit-S	-0.165** (p=0.008)	0.104 (p=0.090)	-0.150* (p=0.015)	0.239** (p=0.000)	0.220** (p=0.000)	-0.198** (p=0.001)	-0.075 (p=0.227)	-0.013 (p=0.832)	0.021 (p=0.736)	0.038 (p=0.546)	0.054 (p=0.384)	0.110 (p=0.076)	-0.017 (p=0.780)	0.087 (p=0.164)
ARS	-0.001 (p=0.993)	0.067 (p=0.067)	-0.187** (p=0.002)	0.058 (p=0.351)	0.033 (p=0.593)	-0.099 (p=0.107)	-0.041 (0.510)	-0.037 (0.554)	-0.125* (p=0.042)	0.011 (p=0.860)	0.024 (p=0.697)	0.103 (p=0.097)	-0.051 (p=0.410)	-0.065 (p=0.297)
DMI (I)	-0.209** (p=0.001)	0.127* (p=0.039)	-0.210** (p=0.001)	0.026 (p=0.671)	0.083 (p=0.184)	-0.043 (p=0.485)	-0.118 (p=0.055)	-0.004 (p=0.955)	0.107 (p=0.083)	0.019 (p=0.758)	0.027 (p=0.659)	-0.013 (p=0.841)	-0.002 (p=0.972)	-0.082 (p=0.189)
DMI (T)	-0.161** (p=0009)	0.175** (p=0.004)	-0.148* (p=0.016)	-0.071 (p=0.251)	0.074 (p=0.233)	-0.061 (p=0.322)	-0.050 (p=0.419)	-0.059 (p=0.341)	-0.079 (p=0.198)	-0.041 (p=0.514)	0.052 (p=0.403)	0.006 (p=0.917)	-0.105 (p=0.089)	-0.087 (p=0.161)

Grit-S – Short Grit Scale, BRS – Brief Resilience Scale, ARS – Academic Resilience Scale, DMI-I– Dweck Mindset Instrument (Intelligence), DMI-T– Dweck Mindset Instrument (Talent)

**correlation is significant at the 0.01 level (2-tailed)

*correlation is significant at the 0.05 level (2-tailed)
